# Concurrent profiling of indole-3-acetic acid, abscisic acid, and cytokinins and structurally related purines by high-performance-liquid-chromatography tandem electrospray mass spectrometry

**DOI:** 10.1186/1746-4811-8-42

**Published:** 2012-10-12

**Authors:** Scott C Farrow, RJ Neil Emery

**Affiliations:** 1 Biology Department, Trent University, Peterborough, ON, K9J 7B8, Canada; 2 Present Address: Department of Biological Sciences, University of Calgary, 2500 University Drive N.W., University of Calgary, Calgary, AB, T2N 1N4, Canada

**Keywords:** Arabidopsis thaliana, HPLC-ESI-MS-MS, Phytohormones, Cytokinins, Abscisic acid, Indole-3-acetic acid, Purines

## Abstract

**Background:**

Cytokinins (CKs) are a group of plant growth regulators that are involved in several plant developmental processes. Despite the breadth of knowledge surrounding CKs and their diverse functions, much remains to be discovered about the full potential of CKs, including their relationship with the purine salvage pathway, and other phytohormones. The most widely used approach to query unknown facets of CK biology utilized functional genomics coupled with CK metabolite assays and screening of CK associated phenotypes. There are numerous different types of assays for determining CK quantity, however, none of these methods screen for the compendium of metabolites that are necessary for elucidating all roles, including purine salvage pathway enzymes in CK metabolism, and CK cross-talk with other phytohormones. Furthermore, all published analytical methods have drawbacks ranging from the required use of radiolabelled compounds, or hazardous derivatization reagents, poor sensitivity, lack of resolution between CK isomers and lengthy run times.

**Results:**

In this paper, a method is described for the concurrent extraction, purification and analysis of several CKs (freebases, ribosides, glucosides, nucleotides), purines (adenosine monophosphate, inosine, adenosine, and adenine), indole-3-acetic acid, and abscisic acid from hundred-milligram (mg) quantities of *Arabidopsis thaliana* leaf tissue. This method utilizes conventional Bieleski solvents extraction, solid phase purification, and is unique because of its diverse range of detectable analytes, and implementation of a conventional HPLC system with a fused core column that enables good sensitivity without the requirement of a UHPLC system. Using this method we were able to resolve CKs about twice as fast as our previous method. Similarly, analysis of adenosine, indole-3-acetic acid, and abscisic acid, was comparatively rapid. A further enhancement of the method was the utilization of a QTRAP 5500 mass analyzer, which improved upon several aspects of our previous analytical method carried out on a Quattro mass analyzer. Notable improvements included much superior sensitivity, and number of analytes detectable within a single run. Limits of detection ranged from 2 pM for (9G)Z to almost 750 pM for indole-3-acetic acid.

**Conclusions:**

This method is well suited for functional genomics platforms tailored to understanding CK metabolism, CK interrelationships with purine recycling and associated hormonal cross-talk.

## Background

Cytokinins (CKs) are a group of plant growth regulators that are involved in several developmental processes, such as: cell division [1], organ source-sink relationships [2,3], and senescence [4,5]. CKs have been implicated in numerous other plant growth and development processes, including long distance signalling [6], and control of stomatal aperture [7]. Moreover, it is now well recognized that the sphere of influence of CKs extends beyond plants into the wider ecosphere including animals, bacteria, fungi and their many pathogenic and symbiotic associations with plants [8].

**Figure 1:**
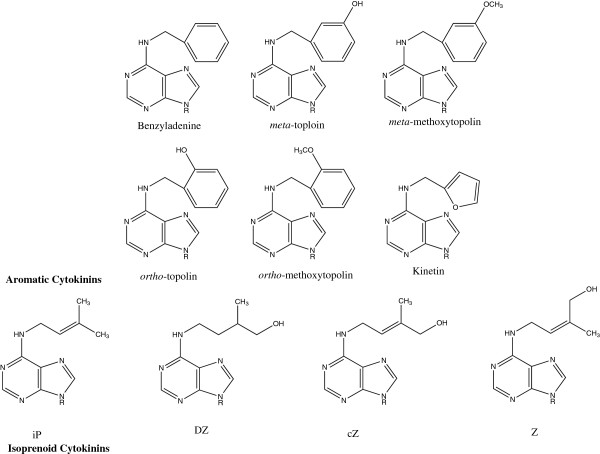
**The structural backbone of some aromatic and isoprenoid cytokinins.** The ‘R’ corresponds to sites of ribosyl and phosphorybosyl functional groups.

**Figure 2:**
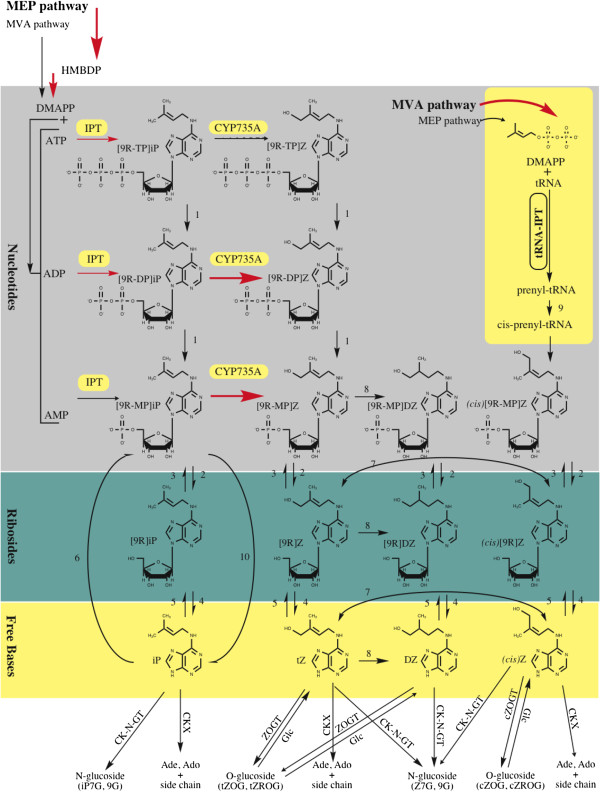
**Proposed cytokinin biosynthesis and metabolic pathways in *Arabidopsis *** **with numbers representing enzymes responsible for the illustrated conversions: (1) phosphatase, (2) 5’-ribonucleotide phosphohydrolase, (3) adenosine kinase, (4) adenosine nucleosidase, (5) purine nucleoside phosphorylase, (6) adenine phosphoribosyltransferase, (7) *cis-trans *** **isomerase, (8) zeatin reductase, (9) *cis*-hydroxylase, (10) LOG phosphoribohydrolase (Adapted from: Sakakibara, 2006 and Kurakawa et al., 2007).**

There are two principal types of CKs – the isoprenoid and aromatic CKs (Figure 1). Much more is known about the more commonly detected, isoprenoid CKs, which are the focus of this study. They are derived from adenine nucleotides, which are prenylated at the *N*^6^ position by either dimethylallyldiphosphate or hydroxymethylbutenyldiphosphate [9,10]. There are four primary base-types of isoprenoid CKs: isopentenyladenine [iP], *cis* and *trans* zeatin (*c*Z and Z), and dihydrozeatin (DZ) (Figure 1). It was suggested that these CKs can be synthesized from two biosynthetic pathways – the iP-derived CKs and the iP-independent CKs [11]. Both starting points yield inactive CK nucleotides (CKNTs), which can undergo conversions to form the purportedly active CKs (Figure 2) – most notably the CK free bases (CKFBs) [12] and, potentially, the CK ribosides (CKRs) [13]. They can also be converted to forms currently considered to be storage or inactive CKs, such as CK *O*-glucosides and *N*-glucosides, respectively [10].

The concentration and type of CKs found in plants at any given time is quite dynamic, particularly considering that CK content changes to meet the demands of the plant, which can change on a short (hours) or long-term (days) basis. For instance, the early developmental, actively dividing tissues consistently have the highest concentrations of CKFBs [14,15]. This is reflected in long-term changes in CK quantity and form such as those seen during the course of seed development and maturation, for which greater concentrations and more active forms are present during times of more active growth [16,17]. Likewise, short-term changes can be observed as CK profiles change sharply, within 1-hour, after manipulations such as apical bud removal [18].

Despite the breadth of knowledge surrounding CKs and their diverse functions, much remains to be discovered about the full potential of CKs. In particular, CK cross-talk with other phytohormones is becoming increasingly important for understanding the complete workings of different plant processes, including meristem development [19], plant osmotic stress responses [20], and plant defence against pathogens [21]. As such, new analytical methods should retain the capacity for simultaneous measurements of as many hormone groups as possible. Furthermore, certain fundamental aspects of CK synthesis and metabolism require authentication [9]. For example, the metabolism of CKs is thought to occur *via* enzymes of the purine salvage pathway (For Reviews see: [9,22-24]). However, this proposition was challenged because of the preference of certain purine metabolic enzymes for purines over CKs in biochemical assays [25-29]. A clear resolution around this issue required a reliable method for the concurrent measurement of CKs and purines, such as the one detailed in this study and which was recently used to study interactions in *Arabidopsis thaliana*[30].

The most widely used approach to query unknown facets of CK biology utilized functional genomics coupled with CK metabolite assays and screening of CK associated phenotypes, and this was demonstrated for the isopentenyltransferase genes [31], CK receptors [32], and the functional analysis of *LONELY GUY*[14]. Previously published analytical assays include: radioimmunoassay [33], enzyme-linked immunosorbent assay [34], gas chromatography (GC)-mass spectrometry (MS)[35], high-performance liquid-chromatography (HPLC)-MS [36], HPLC-MS/MS [37], and ultra-performance liquid-chromatography-MS/MS [38-40]. However, none of these methods screen for the compendium of metabolites that are necessary for elucidating the role of purine salvage pathway enzymes in CK metabolism. Furthermore, all the published methods have drawbacks ranging from the required use of radiolabelled compounds, or hazardous derivatization reagents, poor sensitivity, lack of resolution between CK isomers and lengthy run-times.

Ultra high pressure liquid chromatography (UHPLC) coupled to MS has addressed most of the above drawbacks and has been described as the current standard for CK analysis [38-40]; however, this requires an expensive UHPLC system that can operate at extremely high backpressures. Although this technique is powerful, it is not the only way to achieve fast and sensitive CK analysis. Recently, several chromatographic companies, such as MichromBioresources Inc. (Auburn, CA), Phenomenex (Torrance, CA) and Agilent Technologies (Santa Clara, CA) have developed solid core HPLC columns. Whereas UHPLC columns use small particle sizes (less than 2 μm), creating the drawback of high backpressure and potential column clogging, solid core particles can achieve UHPLC-like performance using a standard HPLC system with benefits of relatively lower backpressure. This technology uses a solid core particle with a thin porous shell that limits the diffusion path of analytes [41].

In addition to offering improvements on, and alternatives to current methods, a goal of this paper was to create an analytical platform that screens for levels of CKs, indole-3-acetic acid (IAA), abscisic acid (ABA), and metabolites of the purine salvage pathway in plants to elucidate the role of purine salvage enzymes, such as adenosine kinase (ADK), in control of CK inter-conversion. ADK is involved in adenosine recycling *via* Ado phosphorylation [27,42,43] and it was thought to facilitate CKR to CKNT conversions [9,26,30,44]. The creation of an analytical platform that simultaneously profiles CK metabolites and those of the purine salvage pathway (i.e. adenosine, adenine, adenosine-5’-monophosphate, inosine) enables a critical tool for investigation of the effects of ADK manipulations, and future CK metabolic gene candidates, on a complete set of metabolites that would be directly affected [30]. Similarly, IAA and ABA are both implicated in CK cross-talk. For example, IAA and CKs have an antagonistic relationship for the control over apical dominance, lateral bud release, and lateral root branching [45], and ABA and CKs coordinate seed germination, nodulation, and senescence [46]. Since, IAA and ABA are the main bioactive forms of these plant hormone types and are fundamental to different plant growth and development processes, an analytical platform that includes IAA and ABA could highlight the presence of, and shed light into the mechanisms of hormonal cross-talk when manipulations are made to key CK metabolic genes.

In this paper we present a rapid, sensitive and cost effective method for the extraction, purification, and analysis of different phytohormone groups including many CKs, IAA, ABA, and compounds that are structurally related to CKs, adenosine (Ado), adenine (Ade), adenosine monophosphate (AMP) and inosine (Ino), without the expense of an UHPLC system. This assay utilizes traditional approaches, such as Bieleski extraction, and solid phase clean-up but is unique in its utilization of a C18 solid core HPLC column coupled to a QTRAP 5500 mass spectrometer and the introduction of an assay for purine compounds. By using this method, we are able to screen for 17 CKs, IAA and ABA, Ade and AMP, Ado, and Ino. This is achievable in less than 15 minutes, which enables processing of close to 100 samples per day. In particular, CK analysis is roughly two times faster than our previous method that used a conventional C18 column [17]. The improvement in sensitivity is over 100 times that of our previous method, which required gram amounts of tissue for CK measurements, along with superior chromatographic resolution. To demonstrate the utility of this method, we applied this analytical system to *Arabidopsis thaliana* plants. This method ensures us that future investigations can accurately quantify a more comprehensive set of phytohormones, and related compounds from 100–300 mg of fresh *Arabidopsis thaliana* leaf tissue.

## Results and discussion

The main objective of this work was to create a high throughput, sensitive and versatile method for the quantification of several CKs, including CKFBs, CKRs, CKNTs, and CK-glucosides, other important plant hormones such as IAA and ABA, and purines, including Ade, Ado, Ino and AMP. All of these compounds needed to be isolated from single 100–300 mg quantity samples of *Arabidopsis thaliana* leaves. By creating this method, an analytical platform for addressing questions related to CK and purine metabolism and associated phytohormone cross-talk is made available. The utility of the method is adaptable for plant systems for which knowledge of phytohormone and/or purine identity and quantity is compulsory. In addition to these requirements, an objective was to create a method that eliminates the dependence upon a UHPLC system.

### Development of hplc-esi(−/+)-Ms/Ms

#### Cytokinin hplc-esi(+)-Ms/Ms

Quantification of CKs has been described previously [37-40,47-52] and some of the important themes discussed in recent work were to separate CK isomers (ie. *c*Z and Z, *(cis)*[9R]Z and [9R]Z) and CK groups (ie. CKFBs and CKRs), achieve high sample throughput, and develop assays capable of detecting a wide range of CKs from small quantities of tissue (< 500 mg).

**Figure 3:**
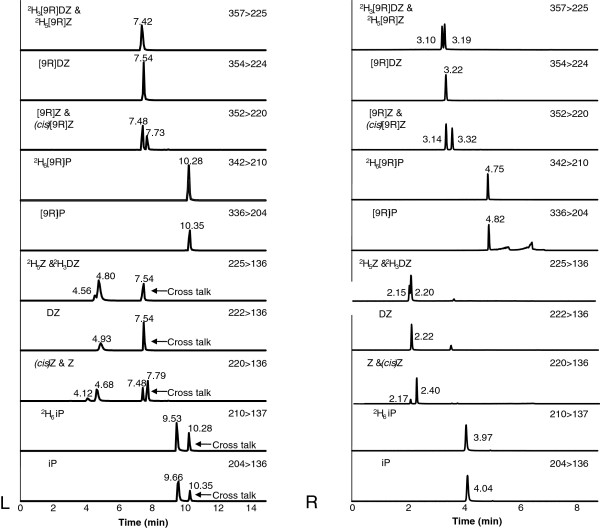
**Quattro® derived chromatogram (L), and QTRAP®5500 derived chromatogram (R) for cytokinin quantification methods.** The run-time and inter-channel cross-talk were reduced, and the peak shape, and resolution improved in the new method.

It is important to resolve CK isomers because these compounds have identical precursor/product ions that cannot be deciphered using MS, and, if not resolved, can result in biased CK quantification [40]. Similarly, many CK forms share common product ions (ie. CKFBs/CKRs), and if certain CK forms are not separated in chromatography space, there is a risk of biased quantification through a phenomenon known as mass spectrometer inter-channel ‘cross-talk’. Inter-channel cross-talk occurs when subsequent multiple reaction monitoring (MRM) transitions sharing common product ions have inadequate inter-channel delay times. To prevent this phenomenon from occurring, subsequent MRM scans must not start until the ions corresponding to the first MRM channel are cleared from the collision cell [53]. The ability of ions to clear the collision cell is limited in standard radio frequency only collision cells; however, we used a mass spectrometer equipped with the Qurved LINAC® collision cell that enables fast scan speeds and markedly reduced cross-talk [51]. The advantage of this technology is faster scan speeds (up to 15,000 Da^.^s^-1^), which results in more sampling points across a chromatographic peak, and can facilitate the quantification of co-eluting compounds that were previously hindered by inter-channel cross-talk. Furthermore, fast scan speeds are an absolute necessity for assays using UHPLC due to very short metabolite elution times inherent with this technology. High sample throughput, assay sensitivity, and assay versatility are a premium for labs that study model plants like *Arabidopsis thaliana*. In particular, the ability to assay small quantities of plant tissue from model systems is becoming increasingly important for understanding spatial/temporal changes in metabolite biosynthesis and metabolism. In our lab’s previous method [17], we were able to separate CK isomers (*c*Z/Z and *(cis)*[9R]Z/[9R]Z) and CK forms (CKFBs from CKRs) using a 15 minute gradient of CH_3_CN and 0.08% CH_3_COOH and a 10 minute re-equilibration period for a total analysis time of 25 minutes (Figure 3). We accomplished this with a 2.1 × 100 mm, 4 μm C18 column, and the assay was capable of detecting and quantifying 11 prominent isoprenoid CKs (iP, *c*Z, Z, DHZ, [9R]iP, *(cis)*[9R]Z, [9R]Z, [9R]DHZ, and the corresponding nucleotide pool of these CKs after dephosphorylation) in several plant systems [17,54,55]; but the method did not completely resolve ^2^H_3_DZR/^2^H_5_ZR and ^2^H_3_DZ/^2^H_5_Z. These analyte combinations suffered from pronounced inter-channel cross-talk (Figure 3), and had low sample throughput, versatility, and sensitivity by today’s standards. More pressing was that the assay was not tailored to small herbaceous model systems like *Arabidopsis thaliana*, which demanded assays capable of monitoring a larger suite of metabolites from lower quantities of tissues.

We recently developed a new CK assay that addresses shortcomings of our previous method and circumvents the requirement of an UHPLC system for high-throughput CK analysis. Our new method resolves CK isomers and CK forms in 8 minutes with a re-equilibration time of 7 minutes (Figure 3) for a total runtime of 15 minutes. The approximately 2-fold improvement in run time facilitates greater sample throughput and the analysis of 4 more isoprenoid CKs than our previous method; moreover, this can all be achieved from 100–300 mg of fresh *Arabidopsis thaliana* leaf tissue. Although recent methods have described short run times and broad CK coverage [38,40], we managed this using a standard HPLC system equipped with a unique core shell Kinetex^©^ C18 column (Phenomenex, Torrence, CA, USA) and the QTRAP 5500 mass spectrometer (AB Sciex, Concord, ON, CA). With this method, we achieved LODs ranging from ≈ 2–10 pM for CKFBs and CKRs, and ≈ 2–20 pM for CK-glucosides, and the %RSD of retention time ranges from 0.2-0.4, where it was 0.86-1.38 in our previous method (Table 1). The LLOQs range from 7–16 pM for CKFBs and CKRs, and 6–72 pM for CK-glucosides (Table 1). This method has the added benefit of using CH_3_OH as opposed to CH_3_CN as the elution solvent, and has reduced inter-channel cross-talk (Figure 3). In addition, we provide MS and methods details for 20 additional CKs for future experiments that need to assay a broader spectrum of CKs (Additional file 1: Table S1 and Additional file 2: Table S2).

**Table 1:** Limits of detection (LOD), limits of quantification (LLOQ), and percent relative standard deviation (%RSD) for cytokinins, purines, abscisic acid and indole-3-acetic acid

**Compound**	**Retention Time (min)**	**%RSD**	**LOD [pM] (3s/m)**	**LOQ [pM] (10 s/m)**
**[**^**2**^**H**_**5**_**](OG)Z**	2.09	0.24	17.71	59.05
**(9G)Z**	2.27	0.24	2.01	6.69
**Z**	2.17	0.17	4.25	14.17
**[**^**2**^**H**_**5**_**](OG)[9R]Z**	2.89	0.17	21.54	71.80
**(OG)[9R]Z**	3.01	0.18	-	-
**[9R]Z**	3.15	0.16	4.18	13.94
***c*** **Z**	2.41	0.31	4.64	15.47
**(*cis*)[9R]Z**	3.33	0.21	3.89	12.98
**[**^**2**^**H**_**7**_**](OG)DZ**	2.26	0.26	7.39	24.65
**DZ**	2.22	0.22	2.13	7.11
**[**^**2**^**H**_**7**_**] (OG)[9R]DZ**	3.08	0.19	16.68	55.59
**[9R]DZ**	3.23	0.15	4.50	15.00
**[**^**2**^**H**_**6**_**](7G)iP**	2.88	0.19	4.69	15.62
**iP**	4.04	0.00	10.16	33.85
**[9R]iP**	4.73	4.93	6.89	22.97
**IAA**	2.04	0.00	742.72	2475.72
**ABA**	2.30	0.00	11.80	39.35
**[**^**13**^**C**_**5**_**]Ade**	2.18	0.01	-	-
**Ade**	2.20	0.00	55.60	185.33
**[**^**13**^**C**_**5**_**]Ado**	0.91	0.53	-	-
**Ado**	0.91	0.00	4.64	15.47
**U**^**15**^**N**_**5**_**Ino**	6.42	0.06	-	-
**Ino**	6.42	0.08	32.54	108.47
**U**^**15**^**N**_**5**_**AMP**	1.87	0.01	-	-
**AMP**	1.89	0.01	51.69	172.28

The analysis of purines *via* HPLC-electrospray ionization (ESI)-positive (+)-MS/MS was described for mammalian samples, including blood [56], urine [57] and other fluids [58]. Purines were assayed from other plant tissues, such as tubers [59] and tea leaves [60]. We wanted to create a more versatile method that permitted the concomitant extraction and purification of purines, several CKs, IAA and ABA from small quantities of *Arabidopsis thaliana* leaf tissue. The availability of this method coupled with functional genomics tools represents a powerful combination for the assessment of the proposed relationship between CKs, purines, and other phytohormones. The assay we developed is capable of detecting and quantifying Ado, Ino, Ade, and AMP from 100–300 mg of *Arabidopsis thaliana leaf* tissue, and the LODs and LLOQs range from 5–60 pM, and 16–200 pM respectively (Table 1), and the %RSD of retention times was 0.17-0.4 (Table 1).

Because purines and phytohormones occur at different physiological concentrations [38,40,60,61], we could not assay purines and phytohormones in one HPLC-ESI(+)-MS/MS run. Purines analysis required dilution before HPLC-ESI(+)-MS/MS, and because Ino, Ade/AMP, and Ado eluted differently from the SPE cartridges, we did not assay all purines simultaneously. By diluting, quantification remained in the linear response range of the MS and this was further controlled by monitoring of internal standards. A persistent advantage to our method is concomitant extraction and purification of CKs, IAA, ABA and purines from the same sample. By assaying a suite of metabolites from the same tissue, possible tissue-to-tissue variations are avoided, and sample preparation time was reduced, which partially alleviated the bottleneck for these assays.

#### Indole-3-acetic acid and abscisic acid hplc-esi(−)-MS/MS

Both IAA and ABA have been assayed using several different techniques including LC-MS-MS [37] and GC-MS [61], and the most common separation and detection system is GC-MS. We have quantified ABA using HPLC-ESI-negative(−)-MS/MS for several years [62,63]; however, IAA had been problematic. Considering the well-established relationship between CKs and IAA [64], and that ABA influences CK dependent processes [9], we wanted to ensure they were included in our assay. IAA is acid labile [38], and we were experiencing IAA degradation in the acidic extraction buffer that was optimized for CKs. We have experienced degradation of ABA in the past, and this was addressed by reducing the exposure of ABA to light during the extraction period. Similarly, we ensured the extraction time for IAA was no longer than 16 hrs, which allowed us to detect and quantify IAA in our assay. The LOD and LLOQ for ABA and IAA were 12/39 and 740/2475 pM respectively, and the %RSD for retention times were 0.16 and 0 (Table 1).

### Quantification of cks, purines, iaa and aba from 100 mg quantities of *arabidopsis thaliana* leaf tissue

We tested the described analytical method on small quantities (100–300 mg) of 4 week old fresh *Arabidopsis thaliana* leaf tissue (n=4), and using this method we were able to detect more forms of CKs than our previous method, it permitted the detection and quantification of several purines, IAA and ABA, peak shape improved for several compounds, and throughput increased by about 2-fold (Table 2). We compared our quantities to other published results for wild type *Arabidopsis thaliana* leaves [65] seedlings [40] and other wild type plants with the hopes of illustrating the analytical robustness of our method and accuracy of the measurements (Table 2). Corbesier et al., [65] and Novak et al., [40] quantified CKs from *Arabidopsis thaliana* leaves and seedlings, respectively, and our results are within the same order of magnitude as their concentration ranges. Small deviations of specific CKs are, of course, likely a result of different tissue types, growing stages, growth conditions, and assay variability. For example, we could not detect DZ in 4 week-old *Arabidopsis thaliana* leaves. The absence of DZ was not surprising considering that DZ is not a major CKFB in *Arabidopsis thaliana*[40,65], its abundance varies with abiotic factors [65], and that CKFBs mostly accumulate in actively dividing tissues like meristems. IAA and ABA concentrations were within the same range as those described by Ghassemian et al., [67], and Kowalczyk and Sandberg, [66], which suggested that our assay was not over or underestimating IAA and ABA quantity. Unfortunately, we could not compare most of our purine concentrations because of a lack of published literature that focused on plants. But Ado/Ade values were roughly 10 times that of ABA and IAA, and up to 1000 times that of certain CKs. AMP was the most abundant purine, and its concentration fell within the range described for fresh and processed tea leaves [60]. It should be noted that these comparisons are not presented with hopes of precise physiological inferences; rather, values from other studies provide a guideline for the analytical robustness and accuracy of the current method. By the same token, if our values deviated greatly from those in the literature, there would be concern about the quantitative utility of our method. We fully expected that different experimental conditions and analytical strategies would result in divergent quantification values.

**Table 2:** **Quantification of endogenous cytokinins, selected purines, abscisic acid and indole-3-acetic acid from *Arabidopsis thaliana *** **leaf**

**Concentration (pmol**^**.**^**g**^**-1 .**^**FW**^**-1**^**)**								
**Metabolite**	This Study (4-week old leaves, n=4)	[40] (10-day old *Arabidopsis* seedlings)	[65] (7 youngest leaves/various organs of *Arabidopsis*)	[66] (*Arabidopsis* leaves)	[67] (*Arabidopsis* plants)	[60] (tea leaves)	[17] (*Pisum sativum* fruits – old method)	[38] (various organs from *Oryza sativa*)
**Z**	4.67 ± 1.92	0.61	0-2	N.M.^a^	N.M.	N.M.	0-25	0-3
***c*** **Z**	6.08 ± 1.03	0.32	N.M.	N.M.	N.M.	N.M.	N.D.	0-12
**DZ**	N.D.^b^	0.02	0-2.5	N.M.	N.M.	N.M.	0-10	N.M.
**iP**	7.65 ± 1.09	0.42	0-2	N.M.	N.M.	N.M.	0-10	0-6
**[9R]Z**	1.19 ± 0.48	2.84	2.5-20	N.M.	N.M.	N.M.	0-25	0-1
***(cis)*** **[9R]Z**	2.42 ± 0.11	1.13	N.M.	N.M.	N.M.	N.M.	50-300	0-10
**[9R]DZ**	0.14 ± 0.07	0.09	0-2	N.M.	N.M.	N.M.	0-20	0-1
**[9R]iP**	1.95 ± 0.39	2.38	2-100	N.M.	N.M.	N.M.	10-100	0-5
**[9RNT]Z**	9.41-NTP^d^ ± 5.27	1.62-MP^e^	4-45-NTP	N.M.	N.M.	N.M.	10-50-NTP	0-2.5
***(cis)*** **[9RNT]Z**	7.42-NTP ± 0.62	N.M.	N.M.	N.M.	N.M.	N.M.	10-200	0-7
**[9RNT]DZ**	N.D.–NTP	N.M.	0-5-NTP	N.M.	N.M.	N.M.	10-50	N.M.
**[9RNT]iP**	13.5-NTP ± 5.24	5.69-MP	15-60-NTP	N.M.	N.M.	N.M.	2-350-NTP	0-19
**(OG)[9R]Z**	3.19 ± 0.31	0.52	N.M.	N.M.	N.M.	N.M.	N.M.	0-0.6
***(cis)*** **(OG)[9R]Z**	3.43 ± 0.31	0.48	N.M.	N.M.	N.M.	N.M.	N.M.	0-50
**(OG)[9R]DZ**	1.31 ± 0.41	N.M.	N.M.	N.M.	N.M.	N.M.	N.M.	N.M.
**(9G)Z**	18.83 ± 2.5	3.72	15-20	N.M.	N.M.	N.M.	N.M.	0-160
**(7G)iP**	N.M.	N.M.	N.M.	N.M.	N.M.	N.M.	N.M.	0-0.2
**IAA**	749.76 ± 111	N.M.	N.M.	28-143	N.M.	N.M.	N.M.	10-300
**ABA**	101.58 ± 30	N.M.	N.M.	N.M.	22.7	N.M.	N.M.	70-1000
**Ade**	2039.73 ± 251	N.M.	N.M.	N.M.	N.M.	N.M.	N.M.	N.M.
**Ado**	1742.93 ± 188	N.M.	N.M.	N.M.	N.M.	N.M.	N.M.	N.M.
**Ino**	725.00 ± 343.5	N.M.	N.M.	N.M.	N.M.	N.M.	N.M.	N.M.
**Amp**	24988.84 ± 4702	N.M.	N.M.	N.M.	N.M.	15000-57000^c^	N.M.	N.M.

We compared our values to our old method and with those from the literature to demonstrate the robustness and accuracy of our method. It should be noted that differences could be due to tissue type, growth stage, growth conditions, and not the analytical techniques.

^a^N.M. = Not Monitored; ^b^N.D. = Not Detected; ^c^ = expressed as (pmol^.^ g^-1.^ DW^-1^); ^d^NTP = nucleotide pool; ^e^MP = CK-monophosphat.

### Core-shell technology as an alternative to a new HPLC system

Over the past five years, sub-2 μm particle size analytical columns have become popular tools for high-throughput quantification. These columns offer improvements in sample throughput, increased sensitivity, and reduced consumption of costly mobile phase solvents like CH_3_CN; however, sub-2 μm packed columns have the inherent drawback of high back-pressures (≈1000 bar). Considering that most standard HPLC systems have an upper backpressure limit of 400 bar, adopting sub-2 μm column technology would require purchasing a new HPLC system. As an alternative to purchasing an UHPLC system, advances in column technology, such as monolithic or core shell columns, have provided avenues for analytical accomplishments that are comparable to UHPLC and with the added benefit of relatively low backpressures [68]. We utilized the core shell column on our standard HPLC system to assay CKs, and we were able to improve several aspects of our CK bioassays, including sample throughput, resolution, and sensitivity.

### Development of solid phase extraction of inosine

Ino is an important nucleoside with varying functions. Ino contributes to the cellular pool of nucleosides, thereby, contributing to whole plant energy charge. It also provides the backbone for biologically important phosphate moieties, which together form the energetically viable plant purines — the nucleotides (ATP, ADP, AMP, IMP; 23, 43). Examples of Ino nucleotides are those common in tRNAs and those which play essential roles in the translation of the genetic code *via* wobble base pairing [68]. Ino has a further function in that it serves as a signaling molecule in the purine salvage pathway. For example, Ado is one of two byproducts of S-adenosylhomocysteine hydrolysis and high levels of Ado can cause inhibition of S-adenosylhomocysteine hydrolysis, which halts S-adenosylmethionine-dependent transmethylation reactions [69]. Ino, on the other hand, is an intermediate of the purine salvage pathway that is routinely sent to the purine catabolic pathway [23].

ESI is an electrical process that is dependent upon the sample matrix, and, therefore, it is important to remove interfering components from the sample prior to analyte analysis. Solid phase extraction of Ino has been described recently [70], and our attempts to purify Ino with Oasis MCX resulted in Ino loss in the flow through and wash steps of our MCX protocol (Additional file 3: Figure S2a). We tested different pH values to improve Ino retention and subsequent purification; however, we were unable to achieve adequate retention behaviour and Ino purification (data not shown). As such, we developed a further SPE protocol, which utilized a mixed anion exchange cartridge (Oasis MAX). Using this protocol, we were able to get better retention and better purity (Additional file 3: Figure S2b).

### Metabolite quantification and calibration curves

#### Quantification

Quantification of metabolites was accomplished using the stable isotope dilution method with the addition of the following labelled standards to each sample after the addition of extraction buffer: 1 ng of each deuterated CK — [^2^H_6_]iP, [^2^H_6_] [9R]iP, [^2^H_6_] [9R-MP]iP, [^2^H_6_] [7G]iP, [^2^H_5_]Z, [^2^H_3_] [9R]Z, [^2^H_6_] [9R-MP]Z, [^2^H_5_] [9G]Z, [^2^H_5_] (OG)[9R]Z, [^2^H_3_]DZ, [^2^H_3_] [9R]DZ, [^2^H_6_] [9R-MP]DZ, and [^2^H_7_] (OG)[9R]DZ — 145 ng of [^2^H_4_] -ABA, 90 ng of [^13^C_6_] IAA, and 250 ng of each purine - [^13^C_5_]Ado, [U^15^N_5_]Ino, [U^15^N_5_]AMP, [^13^C_6_]Ade. Because labelled *c*Z, *(cis)*[9R]Z, *(cis)*[9R-MP]Z and *(cis)*(OG)[9R]Z are not commercially available, the quantification of these compounds was estimated based on the recovery of the isotopically labelled trans-isomers. Greater amounts of ABA, IAA and purine internal standards were added to samples to better match the greater abundances of the naturally occurring counterparts.

#### Calibration curves

Calibrations curves were generated for CKs, IAA, ABA and purines using solutions containing these compounds at different concentrations (5 pM-50 nM). Calibration curves were plotted according to the known concentration of compounds and the measured area under the chromatographic peak (Peak Area Counts; Additional file 4: Figure S1). The resulting calibration curves were used to calculate LODs (defined as 3 times the standard deviation of eight blank responses within the retention time of each analyte), LLOQs (defined as ten times the standard deviation of eight blank responses within the retention time of each analyte divided by the slope of the line), and linearity ranges for each compound (Table 1 and Additional file 1: Table S1). The linear ranges for most compounds spanned four orders of magnitude; however, the dynamic range could be improved using curve fitting or logarithmic transformation.

### Recovery of internal standards

The recovery of labelled standards from plant extracts was estimated using the calibration curves (Table 3). The recovery of CKs varied depending on CK form, and recovery ranged from 10-102%. The recovery of purines ranged from 25-90%, and AMP had the worst recovery. IAA and ABA showed 32 and 39% recovery, respectively. In general, compounds that retained on the MCX cartridge by both the ion and reverse phase mechanisms had the best recovery. Alternatively, CK freebases, nucleotides, IAA and ABA had the worst recovery. Poor recovery is likely attributed to competition for ion exchange and reverse phase sites on the MCX cartridge, and thus, cartridge optimization (i.e. increasing the bed mass) could improve recovery. Alternatively, immunoextraction using a monoclonal antibody with specificity for CKs has been used, and this greatly improved the specificity of recovery and the signal-to-noise ratio in the UV detector and mass spectrometer [40]. While the recovery of more analytes is of interest for improving detection limits, the presence of internal standards in our method accounted for losses and still allowed us to accurately quantify these compounds. However, moving forward, efforts should be made to improve recovery, as this will allow for smaller tissue quantities, perhaps even single cells, to be assayed.

**Table 3:** Percent recovery of internal standards after extraction and purification

**Internal standard**	**% Recovery from plant material**
**[**^**2**^**H**_**5**_**](OG)Z**	70
**[**^**2**^**H**_**3**_**](9G)Z**	75
**[**^**2**^**H**_**5**_**]Z**	10
**[**^**2**^**H**_**5**_**](OG)[9R]Z**	97
**[**^**2**^**H**_**5**_**][9R]Z**	60
**[**^**2**^**H**_**5**_**][9RNT]Z**	10
**[**^**2**^**H**_**5**_**](MeS)Z**	11
**[**^**2**^**H**_**5**_**](MeS)[9R]Z**	78
**[**^**2**^**H**_**7**_**](OG)DZ**	102
**[**^**2**^**H**_**3**_**]DZ**	30
**[**^**2**^**H**_**7**_**] (OG)[9R]DZ**	47
**[**^**2**^**H**_**3**_**][9RNT]DZ**	10
**[**^**2**^**H**_**3**_**][9R]DZ**	83
**[**^**2**^**H**_**6**_**](7G)iP**	82
**[**^**2**^**H**_**6**_**]iP**	43
**[**^**2**^**H**_**6**_**][9R]iP**	28
**[**^**2**^**H**_**6**_**][9RNT]iP**	14
**[**^**2**^**H**_**6**_**](MeS)[9R]iP**	65
**[**^**2**^**H**_**6**_**](MeS)iP**	6
**[**^**2**^**H**_**7**_**]BA**	5
**[**^**2**^**H**_**7**_**][9R]BA**	63
**[**^**13**^**C**_**5**_**] oT**	10
**[**^**13**^**C**_**5**_**]Ade**	75
**[**^**13**^**C**_**5**_**]Ado**	90
**U**^**15**^**N**_**5**_**Ino**	60
**U**^**15**^**N**_**5**_**AMP**	25
**[**^**13**^**C**_**6**_**]IAA**	32
**d**_**4**_**ABA**	39

Percent recovery was calculated using calibration curves.

### Matrix effects

The sample matrix can be a limitation for ESI-MS, and can lead to biased quantification, and unequal comparisons between different plant samples. To assess possible matrix effects we did a spike recovery experiment for all quantified CKs. No significant (P < 0.001) matrix effect could be seen after performing ANCOVA and test for homogeneity of regressions for calibration curves generated with spiked samples vs. calibration curves generated with standards (Additional file 5: Table S3).

### Conclusions and future perspectives

When creating MRM methods, factors such as dwell times, scan speeds, scan segments, and compound ionization are important determinants of the sensitivity of any given mass analyzer. Because certain CKs, and other phytohormones occur at extremely low concentrations, the optimization of these parameters is essential for achieving broad phytohormone detection and quantification. Our method uses generous scan speeds and dwell times, and is capable of detecting a broad range of prominent CKs and purines as well as IAA and ABA. However, optimization of dwell time, scan speed, and the implementation of scheduled MRM that restricts scans to compound elution time will enhance the sensitivity of the assay. One advantage of the QTRAP 5500 is that it has rapid scan speeds (15,000 Da^.^s^-1^), and a greater potential difference across the collision cell which allow for faster clearance of product ions, and thus, reduced cross-talk at even 5 millisecond cycle times. In addition to fast scan speeds, the Turbo-V spray source has very high performance for ionization and transmission into the mass analyzer. The combination of these features, has led to excellent sensitivity [30].

Another advantage to using a QTRAP 5500 mass analyzer is the availability of different scan functions that help validate analytical results. For example, the QTRAP 5500 is equipped with a third quadrupole that can act as a quadrupole ion trap or a mass filter. By simultaneously switching from ion trap to MRM mode, the user can obtain structural information and be more certain of the compound that is eluting [47,71].

The detection of CK nucleotides and glucosides has been described as problematic. For nucleotides, detection was achieved by dephosphorylation of CKNTs to their corresponding CKRs and, as such, this assay favours the detection of the complete CKNT pool, as opposed to determination of each of mono-, di-, and tri-phosphate derivatives. Recently, mono-, di -and tri-phosphate CKNTs have been assayed in their intact forms using HPLC-MS [72], CZE-MS/MS [73], and UPLC®-ESI(−)-MS/MS [37], and a movement towards intact CKNT analysis would improve the versatility and utility of this method for investigations where the concentrations of individual CKNT forms is desired. In this method, CK-glucosides were detected and quantified without problems.

Sample clean-up is important for eliminating matrix components that can interfere with compound ionization. We utilized an SPE protocol developed by Dobrev and Kaminek, [48] and created a new SPE protocol for Ino sample clean-up, which permitted the detection of several compounds of interest. Recently, immunoaffinity purification has been described by Novàk et al., [40] for CKFB, which enhances the detection of CKFBs when present at low levels in plants. This method is beneficial for CK concentration and purification; however, we did not have access to these materials for our analysis.

The analysis of all major phytohormones, including ABA and IAA intermediates, gibberellins, ethylene, brassinosteroids, strigolactones, and jasmonates would expand the scope of our current method, and future work should strive for this. However, because many of these phytohormones can occur at extremely low concentrations, and some do not respond nicely to current mass spectrometer ionization methods, technological improvements in mass spectrometry and sample preparation/clean-up must first be improved before a single method can encompass the profiling of all phytohormone groups.

## Methods

### Reagents and materials

Methanol (CH_3_OH; HPLC grade), acetonitrile (CH_3_CN; HPLC grade), formic acid (HCOOH; 88%), ammonium hydroxide (NH_4_OH; 28%), and ammonium acetate (CH_3_COONH_4_; 97%) were obtained from Fisher Scientific (Ottawa, Ontario, CA). Hydrochloric acid (HCl; 33%) was obtained from J.T. Baker (Phillipsburg, New Jersey, U.S.A.). Ultra-pure water was from a Millipore system (H_2_O; Etobicoke, Ontario, CA). Deuterated CK standards, [^13^C_5_] Ade, authentic CKs and methyl-thio CKs were purchased from OlchemIm Ltd. (Šlechtitelů, Olomouc, CZ). [U^15^N_5_]AMP, [U^15^N_5_]Ino, and [^13^C_6_] IAA were purchased from Cambridge Isotopes (Andover, Massachusetts, U.S.A.). [^13^C_5_] Ado was purchased from Omicron Biochemicals Inc. (South Bend, IN, USA). 5,8',8',8'-, [^2^H_4_] -ABA was purchased from the national research council-plant biotechnology institute (NRC-PBI, Saskatoon, Saskatchewan, CA). Endogenous AMP, Ado, Ade, Ino, IAA, ABA, ethanolamine and bacterial alkaline phosphatase (P4252) were purchased from Sigma Aldrich (Oakville, Ontario, CA). Mixed-mode cation exchange cartridges (Oasis MCX 60 μm, 6 cc, 150 mg), mixed-mode anion exchange cartridges (Oasis MAX 60 μm, 6 cc, 150 mg), and HLB cartridges (Oasis HLB 30 μm, 1 cc, 30 mg) were purchased from Waters (Mississauga, Ontario, CA). PROgene® transport tubes were purchased from Ultident Scientific (St. Laurent, Quebec City, Quebec, CA).

### Biological material

All plant tissue was from facilities at the University of Waterloo using the *Arabidopsis thaliana* (L). Heynh, ecotype, Columbia. Plants were grown on soil containing a 50:50 mixture of Sunshine LC1 Mix and Sunshine LG3 Germination Mix (JVK). Seeds were imbibed in the dark at 4°C for 48 hours before being put under fluorescent light (150 ±20 μmol ^.^m^-2.^s^-1^ photosynthetically active radiation) in an 18 h light/6 h dark photoperiod, set at 21°C. 100–300 mg of *Arabidopsis thaliana* plant leaves were harvested at the 4 week old stage and immediately flash frozen in liquid N_2_ and stored at −80°C until extraction.

### Extraction of cytokinins, abscisic acid, indole-3-acetic acid and purines

**Figure 4:**
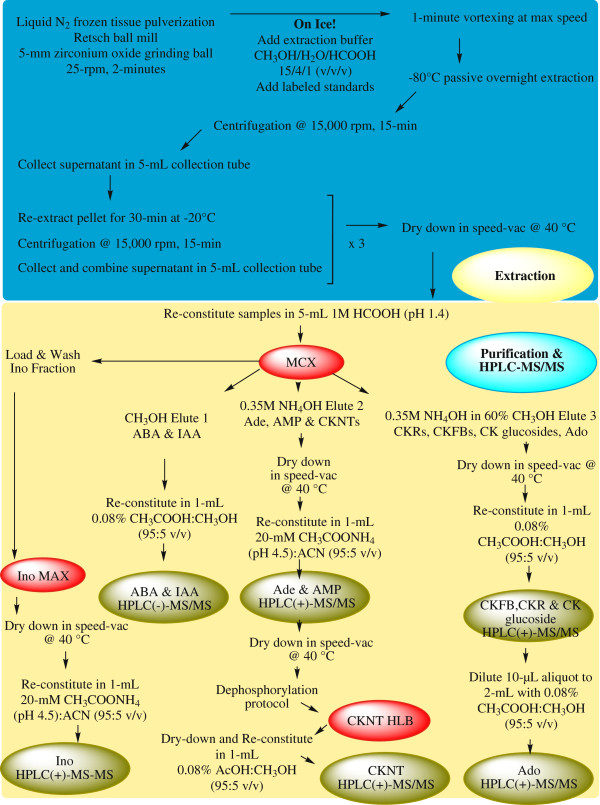
**Extraction, purification, and analytical strategy employed to assay cytokinins, selected purines, abscisic acid and Indole-3-acetic acid from *Arabidopsis thaliana *** **leaves.**

An illustrated summary of the extraction, purification and analysis protocols for all metabolites can be found in Figure 4. CKs, ABA, IAA, and purines were extracted using a modified protocol described by [74] to prevent the enzyme-mediated dephosphorylation of CKNTs and isomerization of CKs. Between 100–300 mg of fresh *Arabidopsis thaliana* leaf tissue was removed from the −80°C freezer, flash frozen in liquid N_2_ and pulverized in a 1.5 mL tube with the assistance of a 5 mm zirconium oxide grinding ball and ball mill (MM 301 Mixer Mill; Retsch GmbH, Haan, DE) set at 25 rpm for 2 minutes. Metabolites were immediately extracted from powdered tissue at 4°C in pre-cooled (−80°C) modified Bieleski reagent (CH_3_OH:H_2_O:HCOOH [15:4:1, v/v/v]) at 5 mL^.^g^-1^ fresh weight (FW) after the addition of stable isotopes, which were used for isotope dilution based quantification. Samples were vortexed for 1 minute and placed in a freezer (−80°C) to allow for passive overnight extraction (≈16 h). Following overnight extraction, solids were removed by centrifugation (4°C, 15,000 rpm, 15 minutes; Eppendorf 5417C, Mississauga, Ontario, CA) and the supernatant was collected in a pre-cooled (−20°C), 5 mL PROgene® transport tube. Solids were re-extracted in modified Bieleski reagent and immediately placed in a −20°C freezer for 30 minutes of passive extraction. Solids were separated by centrifugation and the supernatant was pooled with the first extraction. Solids were re-extracted two more times according to the abbreviated protocol, and pooled supernatants were dried in a speed vacuum concentrator (SPD11V-115, Thermo Electron Corporation, Mississauga, Ontario, CA) set at 40°C, and dried residues were stored at −80°C until solid phase extraction.

### Solid phase extraction of cytokinins, abscisic acid, and indole-3-acetic acid

To purify and concentrate metabolites for HPLC-MS/MS, extraction residues were reconstituted in 5 mL of 1 M HCOOH (pH 1.4) and subjected to MCX solid phase extraction (SPE). The pH of the extract was adjusted between 1.4 - 2.8 with 1 M HCl (2 pH units below the pKa of the most acidic CK), which ensured protonation of CKs and the neutralization of ABA and IAA [48,75]. The pH was monitored with a VWR Symphony pH meter equipped with an epoxy gel combination probe (Mississauga, Ontario, CA). Each extract was purified according to Dobrev and Kaminek, [48] on an MCX SPE cartridge. Cartridges were activated using 5 mL of HPLC grade CH_3_OH and equilibrated using 1 M HCOOH (pH 1.4). After equilibration, the sample was loaded and washed with 5 mL 1M HCOOH (pH 1.4). The elution profiles of the phytohormones can be found in Additional file 3: Figure S2a. The acidic phytohormones eluted first (Elution 1) with 5 mL analytical grade CH_3_OH. CKs were sequentially eluted based on their chemical properties. The CKNTs eluted using 0.35 M NH_4_OH (Elution 2). CKFBs, CKRs, and CK glucosides eluted last using 0.35 M NH_4_OH in 60% CH_3_OH (Elution 3). All samples were evaporated to dryness in a speed vacuum concentrator at 40°C and immediately stored at −80°C until HPLC-ESI(−/+)-MS/MS.

Upon commencing these experiments, CKNTs had never been directly analyzed by HPLC-ESI(−/+)-MS/MS after solid phase extraction. This has been attributed to many factors including poor retention and peak shape on reversed phase columns [72,73]. Moreover, these compounds are relatively unstable if steps are not taken in the extraction process to prevent conversion to their corresponding CKRs [73] or isomerization [74]. It should be noted that CKNTs were recently analyzed directly using UPLC®-ESI(+)-MS/MS and HPLC-ESI(+)-MS/MS [40,73]. In our method, CKNTs were dephosphorylated and detected as their corresponding CKRs. Dephosphorylation was accomplished using 3.4 units of bacterial alkaline phosphatase in 1 mL of 0.1 M ethanolamine-HCL (pH 10.4) for 12 h at 37°C. The resulting CKRs were brought to dryness in a speed vacuum concentrator at 40°C. Samples were reconstituted in 1 mL H_2_O for further purification on a polymeric HLB SPE column (Additional file 3: Figure S2c). Columns were activated with 1 mL HPLC grade CH_3_OH and equilibrated with 1 mL of ultra-pure H_2_O. After column equilibration, samples were loaded and allowed to pass through the cartridge by gravity. The sorbent was washed with 1 mL H_2_O and analytes were eluted using 1 mL CH_3_OH:H_2_O (80:20 v/v). All sample eluents were dried in a speed-vacuum concentrator at 40°C and stored at −80°C until analysis by HPLC-ESI(+)-MS/MS.

### Solid phase extraction of purines

Because purines and CKs share many chemical similarities, the purification protocol implemented for CKs was tested for the Ade, Ado, Ino, and AMP (Additional file 3: Figure S2a). Based on these tests, CKs and purines were purified in one step with the exception of Ino. Ado has two theoretical dissociation constants — pKa 1 = 3.5 (+1) and pKa 2 = 12.3 (0) — and at pH 1.4 Ado retained well on Oasis MCX by both cation exchange and hydrophobicity. As such, Ado elution coincided with CKRs, CKFBs, and CK glucosides (Additional file 3: Figure S2a). Ade has two theoretical dissociation constants — pKa 1 = 4.17 (+1) and pKa 2 is 9.75 (0) — and retains *via* cation exchange as was demonstrated by its elution coinciding with CKNTs (Additional file 3: Figure S2a). AMP has three theoretical dissociation constants — pKa 1= 3.74 (0), pKa 2= 6.05 (−1) and pKa 3= 13 (−2) — and should not retain by cation exchange; however, AMP eluted with the CKNTs, and Ade (Additional file 3: Figure S2a). The retention of AMP has been attributed to electrostatic forces [43]. Ino has three theoretical dissociation constants — pKa 1 = 1.5 (+1) pKa 2 = 8.8 (0) and pKa 3 (−1) = 12.36 — and did not retain well on Oasis MCX at the pH of the loading solvent (pH 1.4), and so a further SPE protocol was developed for Ino. All eluents were dried down under vacuum at 40°C and stored at −80°C until HPLC-ESI(+)-MS/MS.

### Solid phase extraction of inosine

Because Ino did not retain well on the MCX cartridge, we attempted to optimize the loading pH . None of the pH conditions allowed for reproducible retention or purification of Ino, and inhibited concomitant purification of the metabolites of interest (Data not shown). To ensure a clean Ino fraction, the load and washing steps from the MCX protocol were collected and a further solid phase extraction method was developed for Ino. Ino purification was carried out using a Oasis MAX cartridge, which operates best at least two pH units above the pK of the analyte [76]. Ino should have a neutral charge between the pH range of 1.5-8.8 and, therefore, the pH of the loading solvent was adjusted to 11.25 (1.0 M NH_4_OH), to ensure ionization of Ino (−1) and strong binding with the MAX sorbent. The cartridge was activated using 5 mL of CH_3_OH and equilibrated using 5 mL of 1.0 M NH_4_OH (pH 11.25). After Ino had passed through the cartridge by gravity the column was washed with 5 mL of 1.0 M NH_4_OH followed by 5 mL of HPLC grade CH_3_OH. To determine the best elution strength, we tested several combinations of CH_3_OH and HCOOH (Additional file 3: Figure S2b). 1.0 M HCOOH in 50% CH_3_OH was chosen as the elution solvent for Ino, as it allowed for faster drying times in the speed vacuum concentrator. After purification, the Ino fraction was dried down under vacuum at 40°C and stored at −80°C until analysis by HPLC-ESI(+)-MS/MS.

### Instrumentation

For metabolite analysis, a Dionex Ultimate 3000 binary HPLC system (Bannockburn, Illinois, USA) was connected to a QTRAP 5500 triple quadrupole mass analyzer equipped with a turbo V-spray source (ABSciex, Concord, Ontario, CA) operating in ESI(+) mode for CKs and purines, and ESI(−) mode for IAA and ABA. Samples were analyzed with the assistance of the Dionex auto-sampler, and all data were processed by Analyst® quantification software (version 1.5).

### ESI(+/−)-MS/MS conditions

To optimize MS conditions, a 25 ppb solution of each compound was infused into the mass spectrometer at 10 μL^.^min^-1^ with the built-in syringe pump. Optimization of MS conditions, such as declustering potential (DP), and collision energy (CE), was done both manually and automatically using Analyst® compound optimization software, and both methods produced excellent results; however, it should be noted that compound optimization software did not select the optimum diagnostic ions when infusing a complex mixture of CKs. A complete list of MS parameters and diagnostic MRM transitions can be found in Table 4.

**Table 4:** Mass spectrometry parameters used to quantify cytokinins, selected purines, abscisic acid and Indole-3-acetic acid

**Compound**	**Dwell (msec)**	**Declustering Potential**	**Collision Energy**	**Collision Cell Exit Potential**	**Q1 Mass (Da)**	**Q3 Mass (Da)**	**Other Product Ions/(Abundance Rank)**	**Polarity**
**[**^**2**^**H**_**5**_**](OG)Z**	10	116	29	20	387	206.9	225.2 (2),136.8 (3), 135.8 (4), 119 (5)	+
**(OG)Z**	10	75	27	20	382.2	220.1	-	+
**(9G)Z**	10	96	31	14	382	220	135.9 (2), 202 (3), 148 (4), 119 (5)	+
**[**^**2**^**H**_**5**_**]Z**	10	80	32	13	225.1	136.1	119 (2), 207.2 (3)	+
**Z**	10	60	22	11	220.1	136.1	119 (2), 91.3 (3)	+
**[**^**2**^**H**_**5**_**](OG)[9R]Z**	10	71	29	38	519.1	386.9	224 (2), 207 (3), 136.7 (4)	+
**(OG)[9R]Z**	10	71	29	38	514.2	382.2	-	+
**[**^**2**^**H**_**5**_**][9R]Z**	10	58	27	20	357.1	225.2	137 (2), 118 (3)	+
**[9R]Z**	10	60	22	20	352.1	220.2	136.1 (2), 119 (3)	+
***c*** **Z**	10	60	22	11	220.1	136.1	119 (2), 91.3 (3)	+
**(*cis*)[9R]Z**	10	60	22	20	352.1	220.1	137 (2), 118 (3)	+
**[**^**2**^**H**_**7**_**](OG)DZ**	10	76	37	22	390.9	229.1	136 (2), 148 (3), 119 (4)	+
**(OG)DZ**	10	76	37	22	384.2	222.1	-	+
**[**^**2**^**H**_**3**_**]DZ**	10	80	32	13	225.1	136.1	149 (2), 119.9 (3)	+
**DZ**	10	53	29	13	222.1	136	148 (2), 119 (3)	+
**[**^**2**^**H**_**7**_**] (OG)[9R]DZ**	10	71	30	28	523.6	391.2	-	+
**(OG)[9R]DZ**	10	75	31	28	516.2	384.2	-	+
**[**^**2**^**H**_**3**_**][9R]DZ**	10	58	27	20	357.1	225.2	135.9	+
**[9R]DZ**	10	64	25	30	354.2	222.2	136.1 (2), 148.2 (3)	+
**[**^**2**^**H**_**6**_**](7G)iP**	10	56	31	20	372	210.1	137 (2), 119 (2), 74 (3)	+
**(7G)iP**	10	56	31	20	366.2	204.1	-	+
**[**^**2**^**H**_**6**_**]iP**	10	60	22	13	210.1	137.1	148 (2), 119 (3)	+
**iP**	10	50	21	13	204.1	136	148 (2), 119 (3)	+
**[**^**2**^**H**_**6**_**][9R]iP**	10	70	41	15	342.1	137	210 (2), 148 (3), 119 (4)	+
**[9R]iP**	10	74	43	15	336.1	136	204 (2), 148 (3), 119 (4)	+
**[**^**13**^**C**_**5**_**]Ade**	10	96	35	26	140.9	123.8	95 (2). 67 (3)	+
**Ade**	10	96	31	26	136	119	91.8 (2), 64.7 (3), 108.5 (4), 66.7 (5)	+
**[**^**13**^**C**_**5**_**]Ado**	10	35	31	8	272.8	136	118.9 (2), 91.9 (3)	+
**Ado**	10	57	24	18	268.1	135.7	119 (2), 91.9 (3)	+
**U**^**15**^**N**_**5**_**Ino**	10	66	15	8	272.9	140.9	136 (2), 119 (3)	+
**Ino**	10	46	27	16	269.1	136.8	118.9 (2), 68.9 (3)	+
**U**^**15**^**N**_**5**_**AMP**	10	106	29	12	353	140.9	122.9 (2), 69 (3)	+
**AMP**	10	106	25	10	347.9	136	118.8 (2), 96.7 (3), 91.6 (4), 64.8 (5)	+
**[**^**13**^**C**_**6**_**]IAA**	10	40	30	10	180.1	134.2	143.8 (2), 119.1 (3)	-
**IAA**	10	43	26	10	174.1	128	129.7 (2)	-
**d**_**4**_**ABA**	10	50	14	15	267	156.1	223.2 (2),208.1 (3), 98.9 (4), 81.9 (5)	-
**ABA**	10	65	14	7	263	153.1	219.2 (2), 204.2 (3), 203.2 (4), 122.1 (5)	-

Purine HPLC-ESI(+)-MS/MS.

### Turbo-V source optimization

To obtain the highest sensitivity from the mass analyzer, the turbo-V source was flow-optimized by injecting compounds from the syringe pump into a T-splitter at analytical flow rates and with an approximation of the mobile phase constituents that would exist upon compound ionization (50:50 CH_3_OH:0.08% CH_3_COOH for CKs, IAA, ABA and Ado, and 50:50 20 mM CH_3_COONH_4_:ACN (95:5 v/v; pH 4.5):ACN:CH_3_COONH_4_ (95:5 v/v; pH 4.5) for Ade, Ino and AMP). Source parameters such as temperature, ion spray voltage, gas settings, and ESI needle position were optimized for sensitivity. Source conditions for both negative and positive ion modes can be found in Additional file 6: Table S4.

### Quantification

For analytical runs, the effluent was introduced into the Turbo-V spray source using the specific conditions of each metabolite where quantification was obtained by MRM of the protonated [M+H]^+^ or deprotonated [M-H]^-^ intact precursor molecule and a specific product ion. Peaks representing the metabolites were identified according to their retention times, and losses from extraction and purification steps were assumed equal for endogenous and labelled internal standards. Concentrations were calculated by comparing the area under the peak of endogenous compounds with the area under the peak of the corresponding labelled internal standard. The ratio of endogenous to internal standard was used to calculate concentration in pmol^.^g^-1.^FW^-1^. Sample calculation:

A) Concentration = ([100 area counts/1000 area counts] * 1 ng)

B) Concentration = ([0.1/267.241 g^.^mol^-1^] * 1000) /0.5 g

C) Concentration = 0.748 pmol^.^g^.^FW^-1^

### HPLC-ESI(+)-MS/MS of inosine, adenine and adenosine monophosphate

The Ino elution and elution2 (Ade, AMP, CKNTs) were reconstituted in 1 mL of 20 mM CH_3_COONH_4_:ACN (95:5 v/v; pH 4.5) and a 20 μL aliquot was injected onto a Synergi Fusion reversed phase column (4 μm, 2.1 × 150 mm, Phenomenex, Torrance, CA, U.S.A.) for HPLC-(+)-MS/MS. The purines were eluted with an increasing gradient of ACN:20 mM CH_3_COONH_4_ (A; 95:5 v/v,pH 4.5) at a flow rate of 600 μL ^.^min^-1^. The initial conditions were 5% (A) and 95% 20 mM CH_3_COONH_4_ (B; pH 4.5), changing linearly to 40% (A) and 60% (B) in 4 minutes and then immediately to 95% (A) at 4.1 minutes. Conditions remained constant for 1 minute and subsequently changed to initial conditions for 2 minutes yielding a total run time of 7.2 minutes. After analysis, Ade/AMP samples were dried and CKNTs were dephosphorylated for the CKNT HPLC-ESI(+)-MS/MS protocol.

### HPLC-ESI(+)-MS/MS of cytokinins and adenosine

Purified CKNTs and elution 3 (CKFB, CKR, CK-glucosides, Ado) were each reconstituted in 1 mL of the initial mobile phase conditions (0.08% CH_3_COOH:CH_3_OH; 95:5 v/v). For HPLC-ESI(+)-MS/MS, a 20 μL aliquot was injected onto a Kinetex C18 column (2.1 x 100 mm, 1.9 μm solid core, 0.35 μm porous shell; Phenomenex, Torrance, CA, U.S.A.) and compounds were eluted with an increasing gradient of CH_3_OH (A) at a flow rate of 500 μL^.^min^-1^. Initial conditions were 5% (A) and 95% 0.08% CH_3_COOH (B), changing linearly to 45% (A) and 55% (B) in 4 minutes and then changing to 75% (A) and 25% (B) in 5 minutes. At 5.1 minutes, conditions immediately changed to 95% (A) and 5% (B) for 1 minute and then returned immediately to initial conditions at 6.2 minutes for a 2 minute re-equilibration period for a total run time of 8.2 minutes. After the analysis of CKFB, CKR, and CK-glucosides, a 10 μL aliquot was taken and diluted to 2 mL for the analysis of Ado. The same HPLC protocol was used for the analysis of Ado.

### HPLC-ESI(−)-MS/MS of aba and iaa

Elution 1 (ABA and IAA) was reconstituted in 1 mL of the initial mobile phase conditions (0.08% CH_3_COOH:CH_3_OH; 95:5 v/v), and a 20 μL aliquot was injected onto a Kinetex C18 column (2.1 × 100 mm, 1.9 μm solid core, 0.35 μm porous shell; Phenomenex, Torrance, CA, U.S.A.) for HPLC-ESI(−)-MS/MS. ABA and IAA were eluted with an increasing gradient of CH_3_OH (A) at 500 μL ^.^min^-1^. Initial conditions were 5% (A) and 95% 0.08% CH_3_COOH (B) changing linearly to 95% (A) and 5% (B) in 4 minutes. Conditions remained constant for 1 minute and then returned to initial conditions at 5.1 minutes for 2 minutes of equilibration for a total run time of 7.1 minutes.

## Abbreviations

CKs: Cytokinins; CKFB: Cytokinin freebase; CKR: Cytokinin riboside; CKNT: Cytokinin nucleotide; C*K-glucoside*: Cytokininglucoside; iP: Isopentenyladenine; Z: *trans* Zeatin; *c*Z: *cis* Zeatin; DZ: Dihydrozeatin; [9R]iP: Isopentenyladenosine; [9R]Z: *trans* Zeatin riboside; [9R]Z: *cis* Zeatin riboside, (*cis*; [9R]DZ: Dihydrozeatin riboside; [9R-NT]iP: Isopentenyladenosine monophosphate; [9R-NT]Z: *Trans* Zeatinriboside monophosphate; [9R-NT]Z: *Cis* Zeatin riboside monophosphate, (*cis*); [9R-NT]DZ: Dihydrozeatin riboside monophosphate; Ino: Inosine; Ado: Adenosine; Ade: Adenine; AMP: Adenosine monophosphate; IAA: Indole-3-acetic acid; ABA: Abscisic acid; ADK: Adenosine kinase; LOD: Limit of detection; LLOQ: Lower limit of quantification; %RESD: Percent relative standard deviation; UHPLC: Ultra high pressure liquid chromatography; HPLC: High performance (pressure) liquid chromatography; SPE: Solid phase extraction; CID: Collision induced dissociation; MS: Mass spectrometry; ESI: Electrospray ionization; MRM: Multiple reaction monitoring; [M+H]^+^: Protonated Molecular Ion; [M-H]^-^: De-protonated Molecular Ion; HCI: Hydrochloric acid; CH_3_OH: Methanol; CH_3_CN: Acetonitrile; HCOOH: Formic Acid; NH_4_OH: Ammonium Hydroxide; H_2_O: Water.

## Supplementary Material

Additional file 1 **Table S1.** Limits of detection (LOD), limites of quantification (LLOQ), and percent relative standard deviation (%RSD) of 20 additional cytokinins. Click here for file

Additional file 2 **Table S2.** ESI(+)-mass spectrometry parameters for additional cytokinins. Click here for file

Additional file 3 **Figure S2a.** Solid phase extraction of selected purines and cytokinins on Oasis MCX. The purines and cytokinins retained well on MCX accept inosine, which eluted primarily in the load and wash fractions. **Figure2b.** Purification of inosine on Oasis MAX.Inosine retained well via ion exchange mechanisms at pH 11.5 and could be eluted with an acid or acidified CH3OH. **Figure2c.** Purification of cytokininribosides on Oasis HLB after dephosphorylation with bacterial alkaline phosphatase Click here for file

Additional file 4 **Figure S1.** Calibration curves for cytokinins, purines, abscisic acid and indole-3-acetic acid. Click here for file

Additional file 5 **Table S3.** Effect of plant matrix on analyte response in mass analyzer. Click here for file

Additional file 6 **Table S4.** ESI(+/−)-mass spectrometry Turbo-V source conditions used in this study to quantify cytokinins, selected purines, abscisic acid and indole-3-acetic acid. Click here for file
