# Supplementary figures and images for: Concurrent profiling of indole-3-acetic acid, abscisic acid, and cytokinins and structurally related purines by high-performance-liquid-chromatography tandem electrospray mass spectrometry
Source: Plant Methods. 2012 Oct 12;8:42. doi: 10.1186/1746-4811-8-42 (PMC3583190; doi:10.1186/1746-4811-8-42)

A

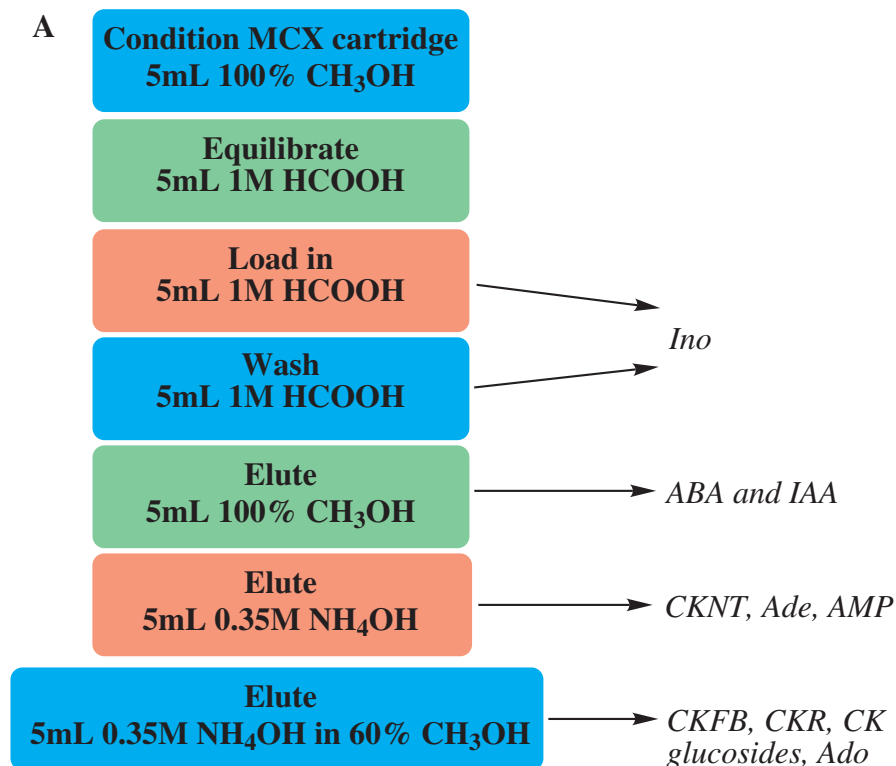

B

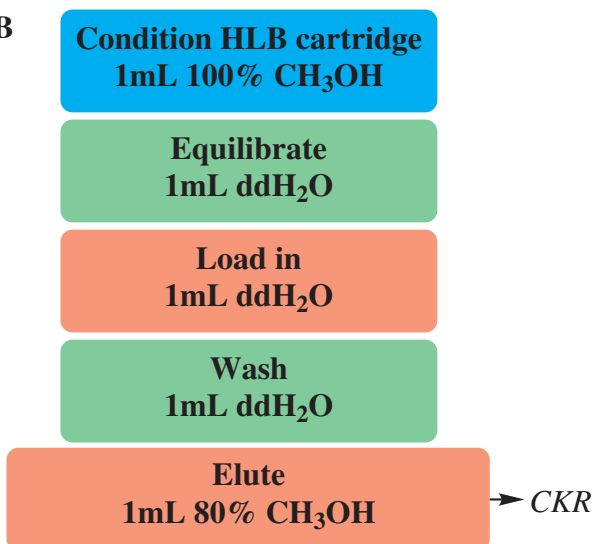

C

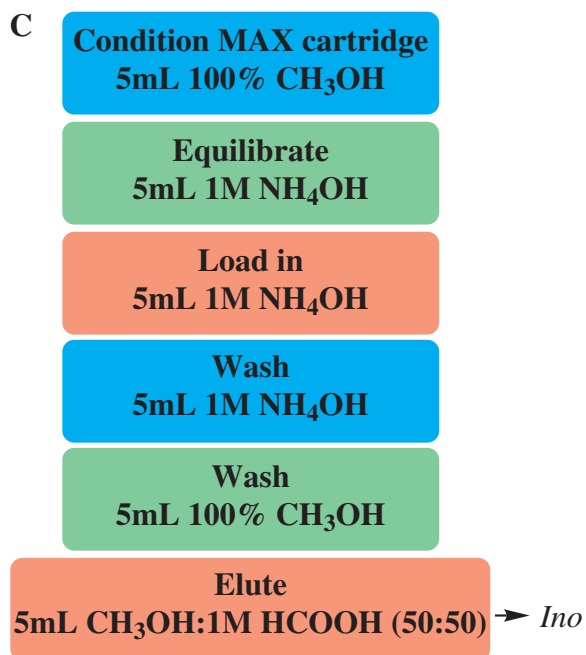

Supplement: Additional file 3 — Figure S2a. Solid phase extraction of selected purines and cytokinins on Oasis MCX. The purines and cytokinins retained well on MCX accept inosine, which eluted primarily in the load and wash fractions. Figure2b. Purification of inosine on Oasis MAX.Inosine retained well via ion exchange mechanisms at pH 11.5 and could be eluted with an acid or acidified CH3OH. Figure2c. Purification of cytokininribosides on Oasis HLB after dephosphorylation with bacterial alkaline phosphatase [file 1746-4811-8-42-S3.pdf]
