# Supplementary material for: Concurrent profiling of indole-3-acetic acid, abscisic acid, and cytokinins and structurally related purines by high-performance-liquid-chromatography tandem electrospray mass spectrometry
Source: Plant Methods. 2012 Oct 12;8:42. doi: 10.1186/1746-4811-8-42 (PMC3583190; doi:10.1186/1746-4811-8-42)
Supplement: Additional file 4 — Figure S1. Calibration curves for cytokinins, purines, abscisic acid and indole-3-acetic acid. [file 1746-4811-8-42-S4.pptx]

## Slide 1
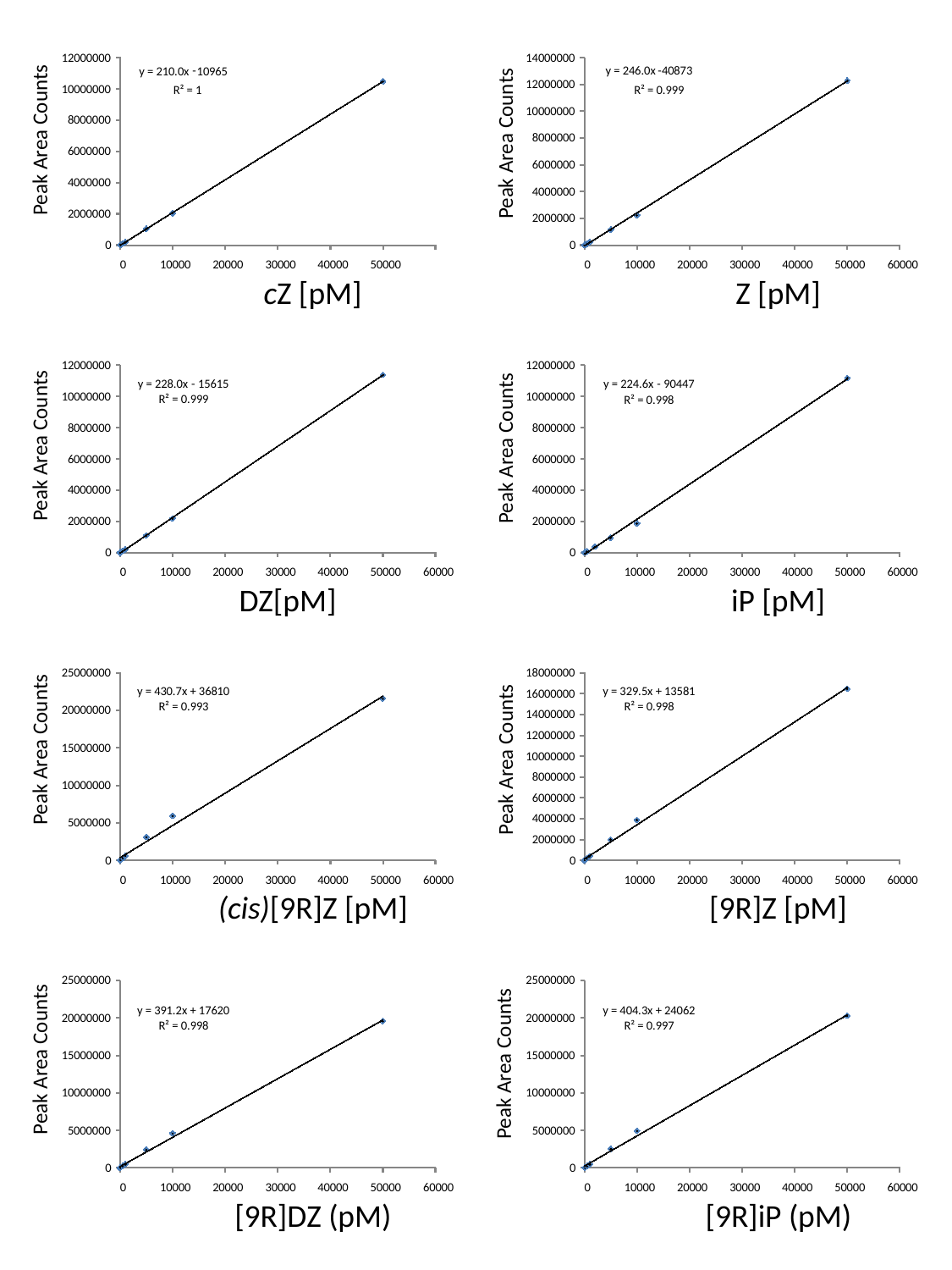

12000000
-
y = 210.0x
10965
R² = 1
10000000
8000000
6000000
4000000
2000000
0
0
10000
20000
30000
40000
50000
cZ [pM]
14000000
y = 246.0x
40873
-
R² = 0.999
12000000
10000000
8000000
6000000
4000000
2000000
0
0
10000
20000
30000
40000
50000
60000
Z [pM]
Peak Area Counts
Peak Area Counts
12000000
y = 228.0x
-
15615
R² = 0.999
10000000
8000000
6000000
4000000
2000000
0
0
10000
20000
30000
40000
50000
60000
DZ[pM]
12000000
y = 224.6x
-
90447
R² = 0.998
10000000
8000000
6000000
4000000
2000000
0
0
10000
20000
30000
40000
50000
60000
iP [pM]
Peak Area Counts
Peak Area Counts
25000000
y = 430.7x + 36810
R² = 0.993
20000000
15000000
10000000
5000000
0
0
10000
20000
30000
40000
50000
60000
(cis)[9R]Z [pM]
18000000
y = 329.5x + 13581
R² = 0.998
16000000
14000000
12000000
10000000
8000000
6000000
4000000
2000000
0
0
10000
20000
30000
40000
50000
60000
[9R]Z [pM]
Peak Area Counts
Peak Area Counts
25000000
y = 391.2x + 17620
R² = 0.998
20000000
15000000
10000000
5000000
0
0
10000
20000
30000
40000
50000
60000
[9R]DZ (pM)
25000000
y = 404.3x + 24062
R² = 0.997
20000000
15000000
10000000
5000000
0
0
10000
20000
30000
40000
50000
60000
[9R]iP (pM)
Peak Area Counts
Peak Area Counts

## Slide 2
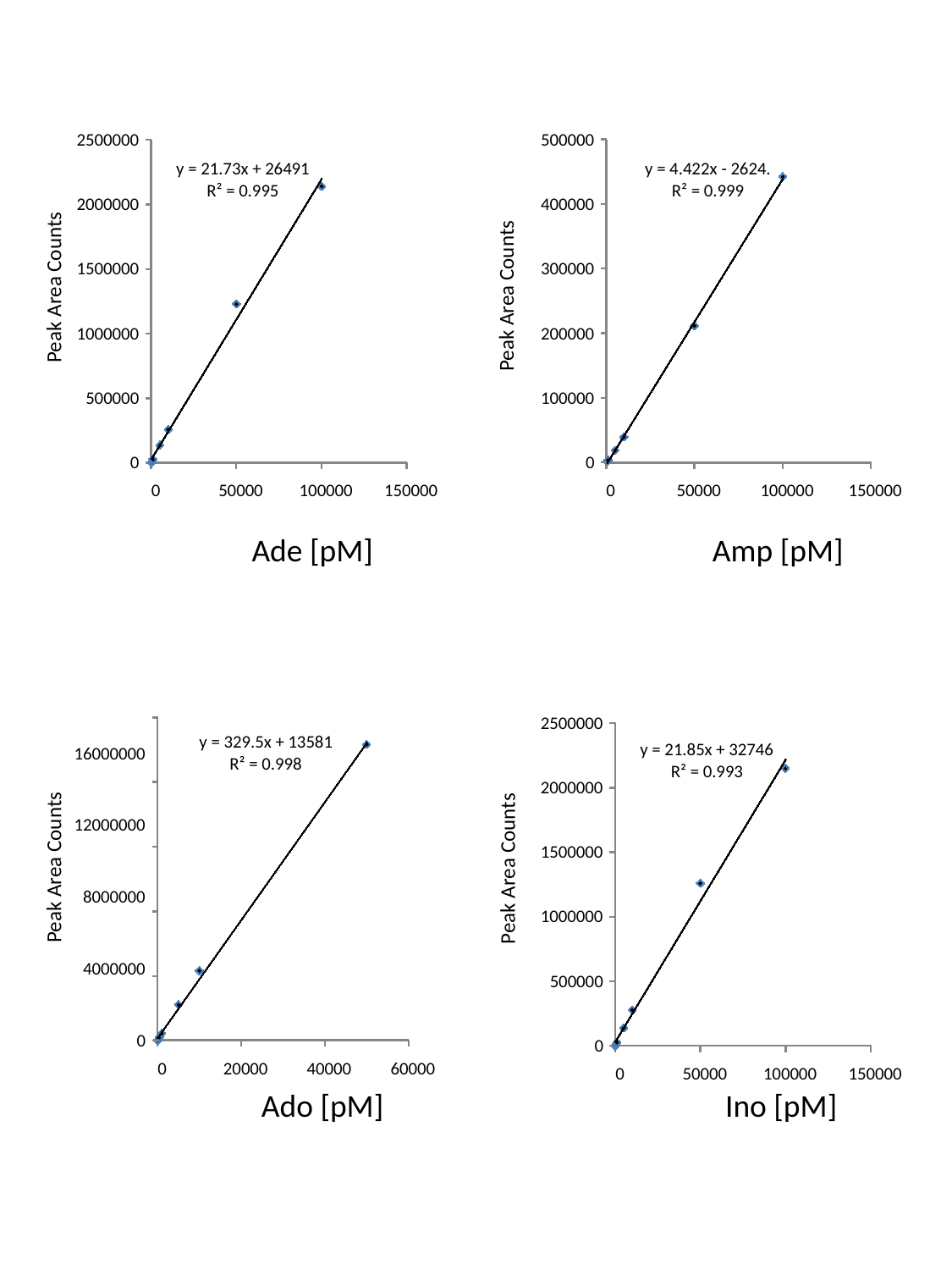

2500000
y = 21.73x + 26491
R² = 0.995
2000000
1500000
1000000
500000
0
0
50000
100000
150000
500000
y = 4.422x
-
2624.
R² = 0.999
400000
300000
200000
100000
0
0
50000
100000
150000
Peak Area Counts
Peak Area Counts
Ade [pM]
Amp [pM]
2500000
y = 21.85x + 32746
R² = 0.993
2000000
1500000
1000000
500000
0
0
50000
100000
150000
y = 329.5x + 13581
16000000
R² = 0.998
12000000
8000000
4000000
0
0
20000
40000
60000
Peak Area Counts
Peak Area Counts
Ado [pM]
Ino [pM]

## Slide 3
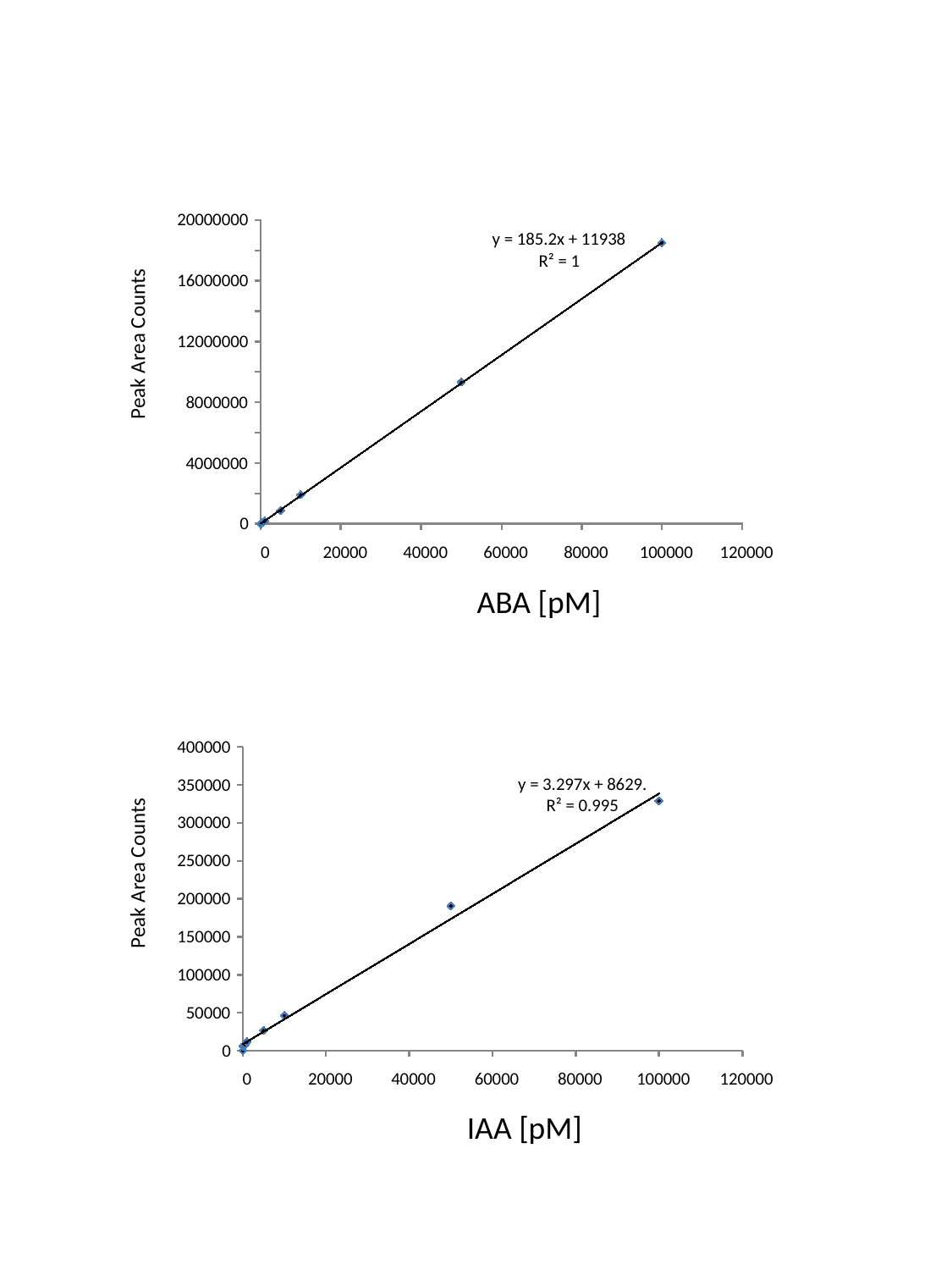

20000000
y = 185.2x + 11938
R² = 1
16000000
12000000
8000000
4000000
0
0
20000
40000
60000
80000
100000
120000
ABA [pM]
Peak Area Counts
400000
y = 3.297x + 8629.
350000
R² = 0.995
300000
250000
200000
150000
100000
50000
0
0
20000
40000
60000
80000
100000
120000
IAA [pM]
Peak Area Counts

## Slide 4
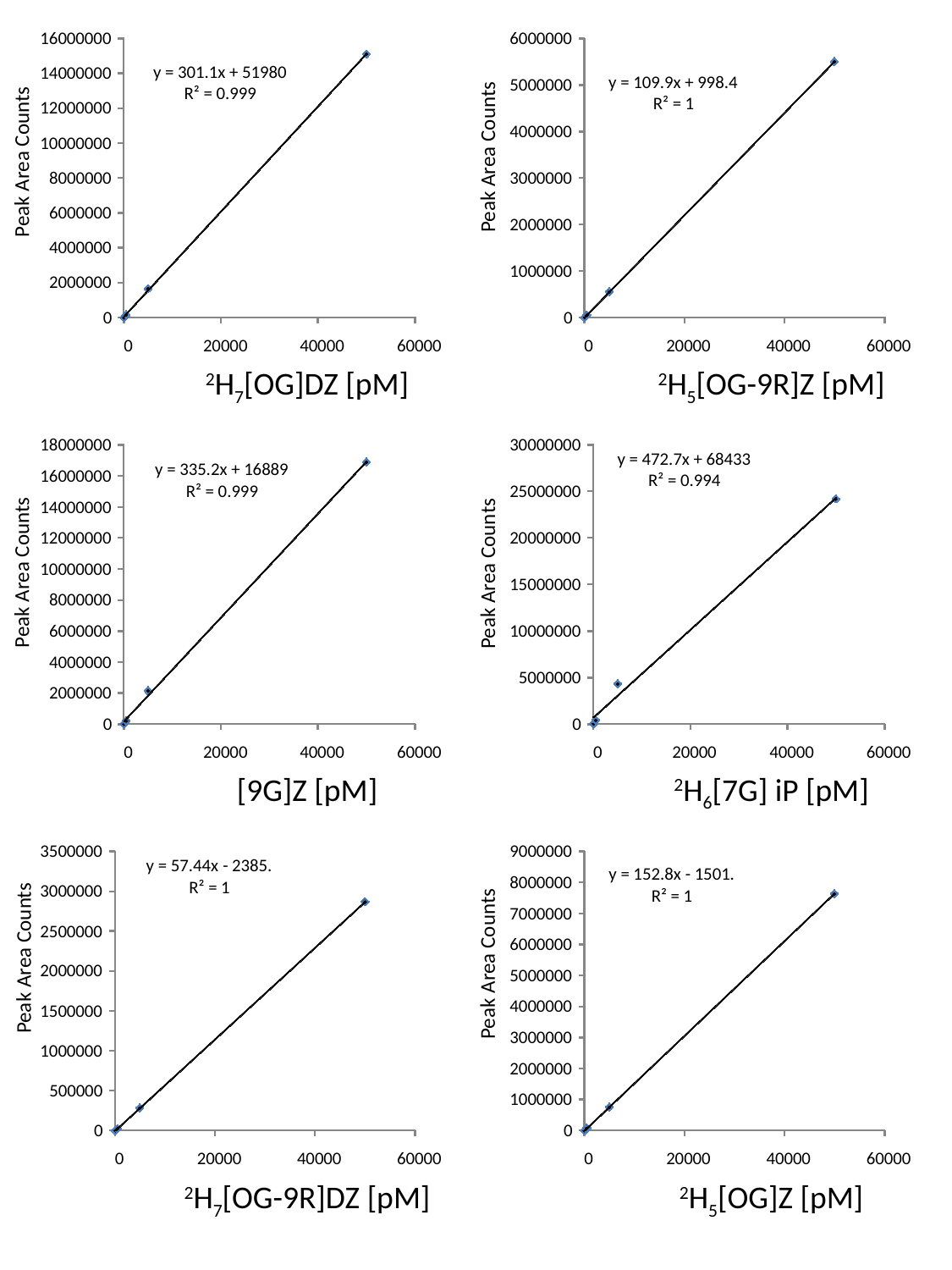

16000000
y = 301.1x + 51980
14000000
R² = 0.999
12000000
10000000
8000000
6000000
4000000
2000000
0
0
20000
40000
60000
6000000
y = 109.9x + 998.4
5000000
R² = 1
4000000
3000000
2000000
1000000
0
0
20000
40000
60000
Peak Area Counts
Peak Area Counts
2H7[OG]DZ [pM]
2H5[OG-9R]Z [pM]
18000000
y = 335.2x + 16889
16000000
R² = 0.999
14000000
12000000
10000000
8000000
6000000
4000000
2000000
0
0
20000
40000
60000
30000000
y = 472.7x + 68433
R² = 0.994
25000000
20000000
15000000
10000000
5000000
0
0
20000
40000
60000
Peak Area Counts
Peak Area Counts
[9G]Z [pM]
2H6[7G] iP [pM]
3500000
y = 57.44x
-
2385.
R² = 1
3000000
2500000
2000000
1500000
1000000
500000
0
0
20000
40000
60000
9000000
y = 152.8x
-
1501.
8000000
R² = 1
7000000
6000000
5000000
4000000
3000000
2000000
1000000
0
0
20000
40000
60000
Peak Area Counts
Peak Area Counts
2H7[OG-9R]DZ [pM]
2H5[OG]Z [pM]
